# Dimensions and position of the eye for facial approximations in a South African cone beam computed tomography sample

**DOI:** 10.1111/1556-4029.15693

**Published:** 2024-12-23

**Authors:** Soné Van der Walt, Anna C. Oettlé

**Affiliations:** ^1^ Anatomy and Histology Department, School of Medicine, Faculty of Health Sciences Sefako Makgatho Health Sciences University Ga‐Rankuwa Gauteng South Africa

**Keywords:** eyeball dimensions, facial approximation, forensic facial reconstruction, ocular position, ocular protrusion, orbital dimensions

## Abstract

Accurate population and sex‐specific normative values for the orbital and ocular dimensions, including the position and protrusion of the eye relative to the orbital rim, are vital for reliable facial approximations. In studies utilizing cadaveric tissue and computed tomography scans, the observed measurements may be influenced by desiccation, distortion or gravity, respectively. This study assessed the dimensions of the eye and orbit and established the position and protrusion of the eye relative to the orbital margin using cone beam computed tomography (CBCT) scans to negate the effect of gravity in the supine position. Scans of 197 adult South Africans (45 Black females, 49 Black males, 55 White females, and 48 White males) were selected retrospectively from private and public hospitals in Pretoria, South Africa. Linear distances were calculated from three‐dimensional landmarks placed on the orbital rim and ocular equator using the MeVisLab © v.3.0.2 software. White females presented with significantly larger orbital heights and axial lengths of the eyes compared to Black females, while the eyeballs of Black females protruded more from the superior and lateral orbital margins. Black females presented with significantly smaller dimensions than Black males. On the contrary, White males exhibited significantly larger protrusion values than White females. The results of this study corroborate with the literature that sex, population, and modality significantly influence the position of the eye in the orbit, which emphasizes the necessity of creating population‐ and sex‐specific facial approximations guidelines for the placement of the eye in the orbit.

**Highlights**

- This study used CBCT scans to accurately determine eyeball position and protrusion.
- Ocular position and protrusion are influenced by population affinity, sex, and modality.
- South Africans have more protruding eyeballs compared to other populations.
- Among South African groups, Black females have the smallest ocular and orbital dimensions.

## 1 INTRODUCTION

The saying, “The eyes are the mirror to the soul” [1], has been interpreted in many ways. Still, in the context of facial approximation, this mirror undeniably assists in facial recognition [2] and is also one of the first features to consider [3, 4, 5, 6, 7, 8, 9, 10].

In the facial approximation process, a standard‐ guideline for eyeball diameter is 25 mm [11, 12]. However, no regard is given to the possible influence of sex or population affinity [12, 13, 14]. In contrast, there are many divergent thoughts regarding the placement of the eye in the orbit. The position of the eyeball in the supero‐inferior and mediolateral planes is sometimes considered to have a central placement within the orbit [6, 15, 16, 17], while other researchers have found that the eyeball is superolaterally located within the orbit [18, 19, 20, 21, 22].

Divergent guidelines regarding the placement of the eyes in the anteroposterior plane or eyeball protrusion, have evolved over the years [2, 7, 21, 23]. Currently, the guidelines propose that the eyeball should be positioned in the bony orbit at a depth at which the iris should touch a tangent taken from the mid superior orbital margin to the mid inferior orbital margin, without taking sex or population affinity into consideration [7, 12, 14, 24].

Facial approximations that rely on absolute measurements based on cadaver studies could be subject to tissue distortion and shrinkage. On the contrary, as gravity has a different effect in the supine position than anticipated for facial approximations viewed in the erect position, measurements derived from scans could be misleading as patients are scanned in the supine position using computer tomography (CT) and magnetic resonance imaging (MRI) [25]. Surprisingly, the literature does not agree on whether gravity significantly impacts soft tissue thickness (STT) [26, 27, 28, 29]. A set of studies confine the positional variations to the lateral facial STT landmarks [25, 26, 27, 30]. However, research specifically examining the effect of gravity on the human eyeball positioning within the bony orbit and surrounding periorbital structures remains limited and not precisely quantified [25, 31, 32, 33, 34].

The position of the eye in the orbit is also a reflection of the ocular and orbital dimensions which may vary between geographically distant groups [7, 22, 35, 36, 37, 38, 39, 40, 41, 42]. Orbital shape variations between population groups may be quantified by determining the orbital index (OI)—the ratio between the orbital height and the orbital breadth [35, 38, 40, 41]. The approximate shape of the orbit may vary from square (OI = 1), rectangular in the horizontal plane (OI <1) to rectangular in the vertical plane (OI >1). Sex and population group variations in ocular and orbital dimensions could therefore have important implications for the positioning of the eyeball and highlight the necessity for population‐ and sex‐specific guidelines.

Despite these reports that inter‐population variation exists [8, 22, 43, 44], guidelines based on other populations are currently used for facial approximations of South African faces [22]. Studies that have relevance to the approximation of the eye based on South African samples are limited. In a pioneering study for the South African context, variations from the published guidelines included: a more rectangular orbit resulting in a more transversely elongated eyeball located superolaterally within the orbit with the exocanthion situated lower than the endocanthion [22]. Unfortunately, the description of the methodology and reported dimensions were not always clear, applicable, and comprehensive and the repeatability was not satisfactory [22]. The small CBCT sample size of only Black South Africans further precluded conclusive comparisons between sexes and population groups.

In this study, we expanded the CBCT scan sample size and incorporated two South African population groups. Landmarks for absolute measurements were revised to improve repeatability and to render it more comparable to other studies. Using CBCT negates the possible effects that desiccation and distortion may have in the case of cadavers and avoids the effect that gravity may have on tissues when in the supine position as noted in CT scanning.

The aim of this study is to determine the dimensions of the eye and orbit in a South African sample and to establish the position of the eye in relation to the orbital margin using CBCT scans. Second, the influence of asymmetry, sexual dimorphism, and population affinity on these distances are determined.

## 2 MATERIALS AND METHODS

### 2.1 Materials

One hundred and ninety‐seven retrospectively collected CBCT scans of adult South Africans without pathological and/or facial deformities and prior surgery to the midface were assessed. The CBCT scans were collected from the Oral Health Centre, Sefako Makgatho Health Sciences University, Oral and Dental Hospital, University of Pretoria, and from a private institution in Pretoria, Republic of South Africa. A Planmeca ProMax CBCT 3D scanner with the following specifications: 90 kV, 8 mA, 11.2 mA, voxel size of 0.4 mm^3^, and a maximum field of view of 230 (diameter) mm by 260 (height) mm was used at the Oral and Dental Hospital, University of Pretoria and the private institution in Pretoria, while a Newtom VGi CBCT 3D scanner with the same specifications and maximum field of view was used at the Oral Health Care Centre, Sefako Makgatho Health Sciences University. The sample consisted of 45 Black females, 49 Black males, 55 White females, and 48 White males aged between 18 and 87 years. Ethical approval to conduct the study was obtained from the Human Research Ethics Committee (HREC) of the Faculty of Health Sciences, University of Pretoria (323/2020).

### 2.2 Methods

The DICOM (Digital Imaging and Communications in Medicine) files were imported into MeVisLab © v.3.0.2 software (available from https://www.mevislab.de/). The MeVisLab software is used for medical image processing and visualization and is based on the “Half Maximum Height” quantitative iterative thresholding method [45]. The segmentation process generates skull and facial surface meshes by separating components based on their gray values [45]. As the density of the eyeball is not different enough from the facial soft tissue structures, and could thus not be reconstructed accurately in three dimensions (3D), the DICOM files were used for the placement of ocular landmarks (Table 1). To prevent orientation bias, the original DICOM files were resliced according to the Frankfort Horizontal (FH) and sagittal planes, as per definition [47], by placing both *porions* and *orbitale* landmarks on the reconstructed skull meshes.

**TABLE 1:** Definition and dispersion (intra‐ and interobserver in mm) of the landmarks.

Landmark	Abbreviation	Definition	Dispersion	
			Intra	Inter
Orbitale	or	Inferior‐most point on the infraorbital margin	L: 0.64 R: 0.86	L: 0.76 R: 1.14
Supraconchion	sk	Superior‐most point on the supra‐orbital margin (excluding the supra‐orbital notch where present)	L: 0.64 R: 0.61	L: 1.22 R: 0.93
Dacryon	d	Junction of the sutures between the frontal, maxillary and lacrimal bones	L: 0.56 R: 0.77	L: 2.66 R: 2.45
Ectoconchion	ek	The most lateral point of the orbital margin following a line bisecting the orbit from the dacryon	L: 0.71 R: 0.61	L: 1.04 R: 1.05
Deepest point on the lateral orbital margin	dLOM	Deepest point on the lateral orbital margin	L: 0.62 R: 0.50	L: 0.66 R: 0.77
Oculus anterius	oa	Most anterior point of the eyeball	L: 0.65 R: 0.67	L: 0.79 R: 0.75
Oculus posterius	op	Most posterior point of the eyeball	L: 0.78 R: 0.93	L: 1.42 R: 1.31
Oculus mediale	om	Most medial point of the eyeball	L: 0.78 R: 0.79	L: 1.20 R: 0.92
Oculus laterale	ol	Most lateral point of the eyeball	L: 0.99 R: 0.89	L: 1.15 R: 1.12
Oculus superius	os	Most superior point of the eyeball	L: 0.93 R: 1.11	L: 1.24 R: 1.28
Oculus inferius	oi	Most inferior point of the eyeball	L: 1.00 R: 1.22	L: 1.30 R: 1.39

*Note*: Landmarks adapted from [2, 39, 46].

### 2.3 Data collection

**FIGURE 1:**
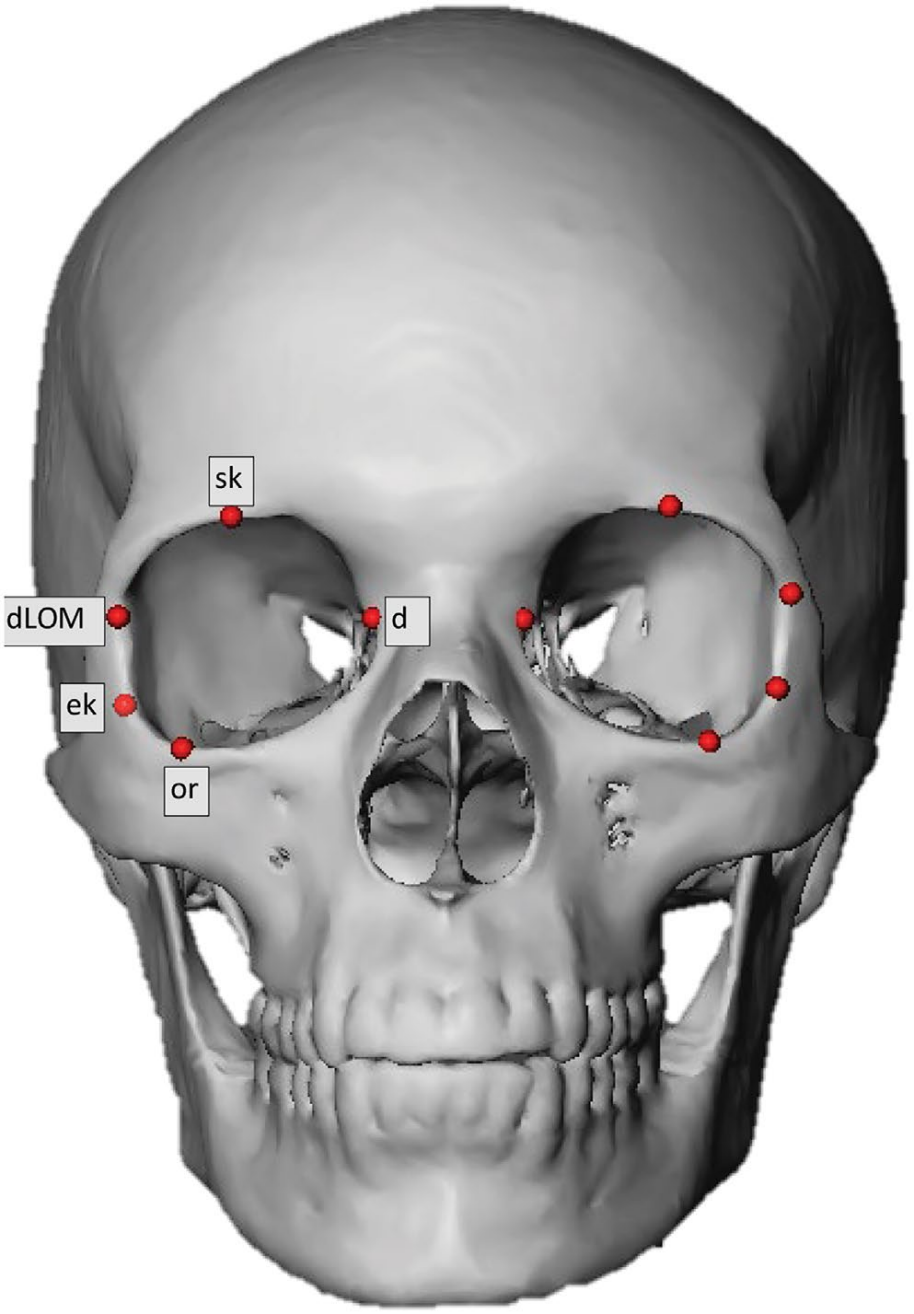
Landmark position on the 3D surface mesh of the skull.

**FIGURE 2:**
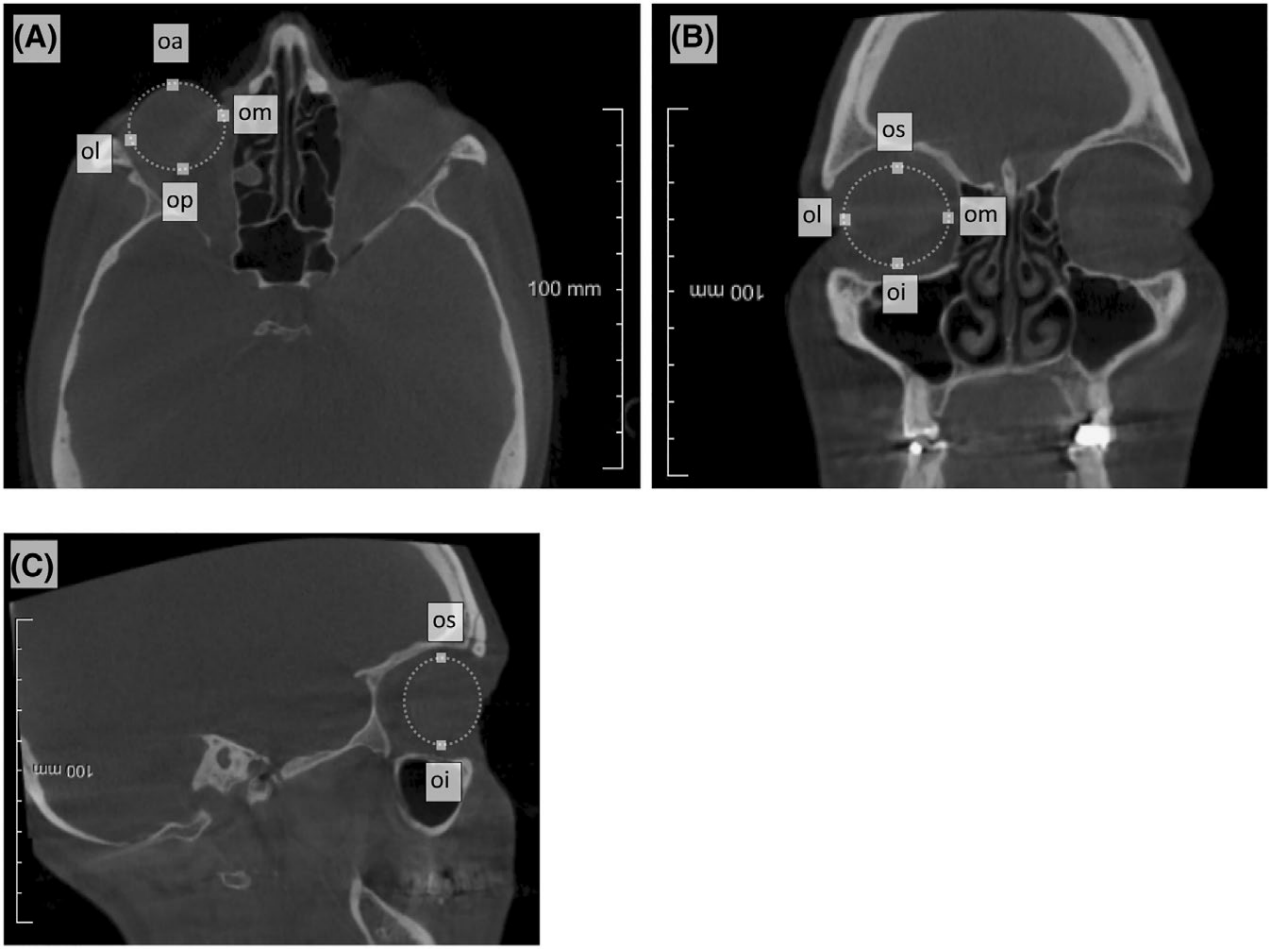
DICOM slice indicating the landmarks used to calculate ocular distances between the white squares. (A) axial view; (B) coronal view; (C) sagittal view.

Landmarks were placed manually on the 3D surface mesh of the skull and 2D DICOM stack (Table 1, Figures 1 and 2), and the 3D interlandmark distances (Table 2) were calculated from the landmark positions using the Pythagorean formula [39]:

$$\text{Distance}\left(a-b\right)=\sqrt{{\left(x_{a}-x_{b}\right)}^{2}+{\left(y_{a}-y_{b}\right)}^{2}+{\left(z_{a}-z_{b}\right)}^{2}}.$$

**TABLE 2:** Measurements and repeatability (ICC values) used in the study.

Measurement	Abbr	Definition	ICC	
			Intra	Inter
Orbital height	sk–or	Distance between the supraconchion and the orbitale	L: 0.91 R: 0.84	L: 0.80 R: 0.89
Orbital breadth	d–ek	Distance between the dacryon and ectoconchion	L: 0.98 R: 0.90	L: 0.63 R: 0.50
Orbital index		(Orbital height/Orbital breadth) * 100	L: 0.91 R: 0.83	L: 0.64 R: 0.42
Ocular height	os‐ oi	Distance between the oculus superius and oculus inferius	L: 0.48 R: 0.29	L: 0.24 R: 0.32
Ocular breadth	ol–om	Distance between the oculus laterale and oculus mediale	L: 0.38 R: 0.58	L: 0.29 R: 0.58
Ocular length (Axial length)	oa–op	Distance between the oculus anterius and oculus posterius	L: 0.66 R: 0.51	L: 0.66 R: 0.32
Superior orbital margin (SOM)—Oculus superius	sk–os	Distance between the supraconchion and the oculus superius	L: 0.82 R: 0.72	L: 0.55 R: 0.73
Inferior orbital margin (IOM)—Oculus inferius	or—oi	Distance between the orbitale and the oculus inferius	L: 0.53 R: 0.62	L: 0.38 R: 0.44
Medial orbital margin (MOM)—Oculus mediale	d—om	Distance between the dacryon and oculus mediale	L: 0.75 R: 0.76	L: 0.16 R: 0.11
Lateral orbital margin (49)—Oculus laterale	ek—ol	Distance between ectoconchion and oculus laterale	L: 0.52 R: 0.71	L: 0.77 R: 0.80
Deepest point of the LOM—Oculus laterale	dlom–ol	Distance from the deepest point on the lateral orbital margin to the oculus laterale	L: 0.62 R: 0.78	L: 0.65 R: 0.62
Eyeball protrusion	dlom–oa	Protruded distance between the deepest point on the lateral orbital margin to the oculus anterius	L: 0.93 R:0.94	L: 0.93 R:0.86
Superior orbital margin (SOM)—Oculus anterius	sk—oa	Distance between the supraconchion and the oculus anterius	L: 0.90 R: 0.80	L: 0.71 R: 0.82
Inferior orbital margin (IOM)—Oculus anterius	or—oa	Distance between the orbitale and the oculus anterius	L: 0.73 R: 0.61	L: 0.83 R: 0.72
Medial orbital margin (MOM)—Oculus anterius	d—oa	Distance between the dacryon and oculus anterius	L: 0.91 R: 0.91	L: 0.55 R: 0.67
Lateral orbital margin (49)—Oculus anterius	ek–oa	Distance between ectoconchion and oculus anterius	L: 0.93 R: 0.89	L: 0.91 R: 0.88

*Note*: Landmarks adapted from [2, 39, 46].

Franklin and colleagues have already pointed out in 2005 that linear measurements derived from traditional anthropometric measuring techniques, are comparable with linear measurements derived from three‐dimensional landmark coordinates and can be successfully used in traditional linear dimension studies [48].

The position of the eye relative to the orbital margin was determined in two different ways so as to (1) maximize its comparison with the literature and (2) its usability for facial approximations. The position of the equator of the eyeball in relation to the orbital margin can be used to measure the depth of the placement of the eye within the orbit, in contrast, the position of the oculus anterius in relation to the orbital margin can be used to define the protrusion of the eye.

### 2.4 Statistical analysis

The PAST v. 4.11 program was used for the statistical analysis of the data [46]. Repeated measures were performed by the principal investigator and an independent researcher to determine the intra‐observer and interobserver reliability. The accuracy of the landmark placement was assessed by performing a dispersion analysis (determining the mean distance between the average position of each landmark in relation to the repeated landmarks) [49]. Results are presented in Table 1. An intraclass correlation coefficient test (Two‐way random effects, absolute agreement, single rater/measurement: ICC (2,1)) was performed to determine the reliability and repeatability of the linear distances (Table 2). Interpretation of the ICC results was based on the description by Koo and Li, 2016 [50].

A Shapiro–Wilk test was used to determine the distribution of the data. Univariate analysis (mean, standard deviation, and range) followed for each sex and population group. To determine the statistical variance between the sex and population groups, an ANOVA test was used for parametric data and a Kruskal–Wallis test for non‐parametric data. In order to address the Family–Wise Error Rate and to prevent Type I errors, ad hoc tests were performed [51, 52, 53, 54]. For the parametric data, a Tukey's Pairwise [52] test was used and for non‐parametric tests, a Dunn's post hoc test was used, with sequential Bonferroni significance [51, 54]. To prevent false significant comparisons due to the small sample size [55], statistical significance was set at 5% (*p* ≤ 0.05) and 0.5% (*p* ≤ 0.005) [56].

To statistically compare the findings of this study to available literature in the discussion section of the paper, a comparative analysis was performed using two‐sample *t*‐tests (*BSDA* package in R) and a Bayes Factor calculation (*BayesFactor* package in R studio) [57, 58]. The Bayes Factor (BF) quantifies the strength of evidence for or against the null hypothesis, which states that there is no difference between population means. A BF between −0.5 and 0.5 indicates weak evidence, 0.5–1 indicates moderate evidence, 1–1.5 indicates strong evidence, 1.5–2 indicates very strong evidence, and a BF greater than 2 indicates decisive evidence. Negative values indicate evidence for the null hypothesis, with the same strength as the corresponding positive values. Due to the extensive nature of the test, the left side was used for comparative purposes (Table S1).

## 3 RESULTS

The average dispersion of the landmarks for the intra‐observer tests was 0.80 ± 0.19 mm and 1.21 ± 0.49 mm for the interobserver tests (Table 1). The intraclass correlation coefficient (ICC) indicated greater repeatability of measurements by the principal investigator (intra‐observer) compared to the independent investigator (interobserver), although agreement followed a similar trend. The mean ICC for the intra‐observer test was 0.73 ± 0.18 and 0.61 ± 0.23 for the interobserver test. Excellent repeatability (ICC >0.9) was noted for the orbital dimensions. The protrusion of the eyeball from the orbital margin could be determined with greater repeatability compared to the position of the eyeball in relation to the orbital rim (Table 2).

All data were normally distributed in the White South African sample, except for the right orbital index and right ocular width in males, while the right orbital breadth and right axial ocular length were non‐parametric in the female group. In Black males, the ocular indexes, sk‐os (right) and d‐oa (left) were non‐parametric, while the remainder of the data was parametric. All linear distances in the Black female sample were parametric, besides the left orbital index, right ocular width, sk‐os, right ek‐oa, and right d‐oa. Table 3 presents the summary statistics of the orbital and ocular dimensions, the position of the eye in the bony orbit, as well as the results of the influence of asymmetry, sexual dimorphism, and population affinity. The effect of sex within population groups and the influence of population affinity within sex groups were further investigated and presented in Table 4.

**TABLE 3:** Univariate analysis of distances in mm and *p* values testing the influence of asymmetry, population affinity and sexual dimorphism of orbital and ocular dimensions.

Measurement	Side	Black female *n* = 45	Black male *n* = 49	White female *n* = 55	White male *n* = 48	Asymmetry (p)	Population (p)	Sex (p)
Orbital height	L	**35.73** *2.98* (29.44–42.22)	**37.76** *2.28* (33.63–45.06)	**37.32** *2.28* (30.52–43.60)	**38.05** *2.15* (33.18–42.78)	0.723	0.016*	0.000**
	R	**35.91** *2.94* (28.74–40.23)	**37.73** *2.69* (32.34–43.93)	**37.62** *2.06* (33.31–42.14)	**37.98** *2.39* (33.34–43.35)		0.012*	0.010**
Orbital breadth	L	**40.10** *2.79* (35.73–46.30)	**42.27** 1.62 (39.10–45.77)	**40.52** *1.45* (37.63–44.60)	**42.76** *1.50* (39.41–45.83)	0.374	0.773	0.000**
	R	**40.14** *2.63* (35.55–45.32)	**42.54** *1.80* (38.10–46.28)	**40.56** *1.68* (38.02–44.46)	**43.51** *2.14* (40.03–48.98)		0.394	0.000**
Orbital index	L	**89.17** *5.09* (81.82–103.14)	**89.36** *5.08* (80.12–106.38)	**92.19** *6.20* (75.74–108.52)	**89.03** *4.66* (77.34–98.01)	0.625	0.030*	0.040*
	R	**89.53** *5.80* (78.84–102.51)	**88.73** *5.72* (77.06–102.25)	**92.87** *5.60* (78.14–106.34)	**87.04** *5.73* (74.36–100.59)		0.028*	0.035*
Ocular breadth (OB)	L	**22.44** *1.60* (19.79–26.43)	**23.34** *1.47* (20.59–26.92)	**23.23** *1.20* (20.65–26.08)	**23.55** *1.39* (20.00–26.99)	0.343	0.022*	0.005*
	R	**22.78** *1.54* (20.02–26.70)	**23.61** *1.47* (20.72–26.47)	**23.14** *1.38* (20.10–25.85)	**23.62** *1.49* (21.29–27.31)		0.497	0.002**
Ocular height (OH)	L	**23.77** *1.44* (19.98–26.40)	**24.60** *1.86* (20.80–28.40)	**23.88** *1.43* (20.80–27.20)	**25.23** *1.55* (22.40–28.80)	0.511	0.182	0.000**
	R	**23.89** *1.66* (20.80–27.85)	**23.92** *1.97* (18.40–28.00)	**23.93** *1.40* (20.80–26.80)	**25.30** *1.47* (22.40–28.40)		0.005**	0.000**
Axial/ocular length (OL)	L	**21.98** *1.23* (18.28–25.10)	**23.14** *1.30* (20.29–25.85)	**23.50** *1.22* (20.54–26.31)	**23.27** *1.17* (20.82–25.68)	0.692	0.000**	0.047*
	R	**22.16** *1.36* (19.22–24.80)	**23.39** *1.04* (20.90–25.85)	**23.27** *1.28* (20.12–26.87)	**23.32** *1.05* (21.26–25.46)		0.007*	0.001**

*Note*: Bold: mean, Italics: standard deviation, Brackets indicates the minimum and maximum values. Statistically significant *p ≤ 0.05, **p ≤ 0.005.

**TABLE 4:** Variation in orbital and ocular linear dimensions between sexes within populations and between populations within sexes.

Measurement	Side	Sexual dimorphism within populations		Population variation within sex groups	
		Black south Africans *n* = 94	White south Africans *n* = 103	SA females *n* = 100	SA males *n* = 97
Orbital height	L	0.000**	0.426	0.007*	0.932
	R	0.003**	0.887	0.005**	0.960
Orbital breadth (d‐ek)	L	0.000**	0.000**	0.926	0.590
	R	0.000**	0.000**	0.839	0.099
Orbital index (OH/OB *100)	L	0.886	0.003**	0.003**	0.946
	R	0.521	0.000**	0.019*	0.180
Ocular height (OH)	L	0.052*	0.000**	0.973	0.181
	R	0.999	0.002**	0.995	0.002**
Ocular breadth/width (OB)	L	0.011*	0.610	0.033*	0.878
	R	0.003*	0.331	0.106	0.999
Axial length/ocular length (OL)	L	0.000**	0.753	0.000**	0.948
	R	0.000**	0.999	0.000**	0.759

*Note*: Statistically significant *p ≤ 0.05, **p ≤ 0.005.

Significant asymmetry was noted in the position of the eye with regard to the following measurements: LOM‐ol (*p* = 0.002) and LOM‐oa (*p* = 0.000). The position of the eyes was shifted to the left as the right eye was positioned closer to the medial orbital margin (MOM) (mean difference: 0.67 mm) on the right, while closer to the left orbital margin (ectoconchion) (mean difference: 0.55 mm) on the left. As expected, the anterior‐most point of the eye (oa) was also closer to the left orbital margin (ectoconchion) (mean difference: 0.97 mm) compared to the right.

The orbital breadth was consistently greater than the orbital height in all four South African sex‐population groups suggesting a rectangular‐shaped orbit in the horizontal plane, although a smaller variation was noted in White females, suggesting a more square‐shaped orbit in this group (Table 3). Although the orbital height was significantly larger in White as compared to Black South Africans and males as compared to females, the orbital breadth was sexually dimorphic, but not population‐specific. With further investigation with regard to variation among sex‐population groups, it was noted that orbital height and breadth are sexually dimorphic in Black South Africans, while only orbital breadth varied between White males and females, which resulted in a greater orbital index of White South African females than the other sex‐population groups (Table 4). A more profound effect of population affinity was noted when sex groups were considered in isolation compared to the entire population. Similar orbital dimensions were observed in South African males, while Black South African females had significantly smaller orbital dimensions compared to White South African females.

Significant population variation could be noted in the ocular dimensions of South Africans, as recorded in Table 3. Black South African females presented with significantly smaller ocular dimensions compared to their male counterparts and White South African females (Table 4).

Based on the distances between the orbital margin and the equator of the eye, it was noted that the equator of the eyeball was located significantly deeper within the bony orbit in White South Africans, compared to Black South Africans in relation to the superior, inferior and medial orbital margins (Table 5). Sex‐ and population‐specific variations in the dimensions of the eyeball and orbit had a direct influence on the position of the eyeball resulting in greater variation in the ocular position in females compared to males (Table 6). Greater orbital heights in White females compared to Black females, lead to greater distances from the superior and inferior orbital margins to the equator of the eye. Sexual dimorphism in Black South Africans was observed in the position of the eye in relation to the superior orbital margins, while sex influenced the position in White South Africans in the horizontal plane only, which could be due to significantly greater orbital breadths noted in White South African males.

**TABLE 5:** Univariate analysis of distances in mm and *p* values testing the influence of asymmetry, population affinity and sexual dimorphism of ocular position and protrusion.

Measurement	Side	Black female *n* = 45	Black male *n* = 49	White female *n* = 55	White male *n* = 48	Asymmetry (p)	Population (p)	Sex (p)
SOM‐os	L	**9.40** *1.96* (6.24–13.76)	**10.64** *1.84* (6.06–14.35)	**11.98** *1.68* (7.59–16.08)	**11.78** *2.26* (6.80–17.30)	0.211	0.000**	0.218
	R	**9.53** *1.40* (7.36–14.03)	**11.20** *2.17* (8.04–17.32)	**12.28** *1.76* (8.12–17.31)	**12.28** *2.20* (7.58–18.73)		0.000**	0.047*
IOM‐oi	L	**8.99** *1.80* (5.30–11.89)	**9.04** *2.22* (4.85–14.63)	**10.38** *1.83* *(5.44–14.68)*	**9.60** *1.30* (6.66–12.61)	0.636	0.000**	0.107
	R	**8.93** *2.18* (4.93–13.58)	**9.23** *20.3* (5.06–15.41)	**10.12** *1.50* (7.02–13.45)	**9.38** *1.76* (5.29–12.98)		0.011*	0.300
MOM‐om	L	**11.57** *1.45* (8.74–14.57)	**11.97** *1.61* (7.99–14.76)	**11.81** *1.45* (9.30–15.45)	**13.13** *1.42* (10.32–16.44)	0.020*	0.004**	0.000**
	R	**10.70** *1.29* (8.04–14.61)	**12.08** *1.75* (80.2–15.69)	**11.81** *1.45* (8.68–14.72)	**12.67** *1.67* (9.37–15.64)		0.012*	0.000**
LOM (ek)‐ol	L	**11.84** *1.85* (7.42–15.43)	**10.86** *1.34* (7.82–13.59)	**10.44** *1.55* (7.32–14.08)	**12.74** *1.62* (8.88–16.59)	0.002**	0.482	0.006*
	R	**12.01** *1.63* (9.29–15.43)	**10.94** *1.67* (7.64–14.91)	**11.80** *1.80* (6.68–16.12)	**13.31** *1.35* (10.67–16.31)		0.000**	0.402
LOM (dLOM)‐ol	L	**9.39** *1.96* (5.92–14.70)	**9.17** *1.81* (5.44–13.37)	**8.29** *1.68* (4.70–12.34)	**9.30** *1.98* (5.60–13.89)	0.592	0.057	0.096
	R	**9.45** *11.69* (5.09–13.94)	**9.07** *1.84* (5.99–13.46)	**8.23** *1.50* (4.99–11.37)	**9.65** *1.49* (6.38–12.30)		0.142	0.018*
Protrusion (dLOM—oa)	L	**24.02** *2.26* (19.15–28.34)	**24.60** *1.91* (20.44–28.10)	**23.59** *1.61* (20.55–27.92)	**25.16** *1.81* (21.71–28.98)	0.080	0.991	0.000**
	R	**24.17** *1.91* (20.18–29.62)	**24.31** *1.89* (20.35–28.93)	**22.89** *1.97* (18.78–26.72)	**24.66** *1.90* (20.95–29.13)		0.066	0.000**
SOM‐oa	L	**21.31** *2.74* (15.71–26.43)	**20.84** *2.04* (16.46–26.61)	**19.59** *2.06* (14.55–24.73)	**19.65** *1.85* (15.87–23.62)	0.942	0.000**	0.725
	R	**20.95** *2.51* (14.84–25.13)	**21.00** *2.12* (16.25–26.64)	**19.58** *2.15* (13.95–24.45)	**19.60** *2.05* (14.09–23.32)		0.000**	0.729
IOM‐oa	L	**20.51** *2.90* (15.26–26.70)	**19.70** *1.99* (15.88–25.08)	**20.13** *1.80* (15.96–23.79)	**20.42** *1.75* (17.05–24.80)	0.069	0.480	0.276
	R	**19.76** *2.59* (14.73–24.80)	**19.00** *1.96* (14.59–23.69)	**20.08** *2.10* (16.01–24.45)	**19.92** *2.01* (15.86–25.26)		0.039*	0.116
MOM‐oa	L	**23.35** *2.47* (18.63–27.85)	**23.20** *1.97* (16.59–26.65)	**22.81** *1.67* (19.12–26.95)	**24.33** *1.71* (21.53–28.33)	0.266	0.394	0.014*
	R	**24.70** *3.93* (19.03–35.31)	**22.89** *1.90* (17.71–27.30)	**22.57** *2.02* (18.61–26.92)	**23.66** *1.94* (19.68–29.08)		0.308	0.495
LOM‐oa	L	**23.79** *1.53* (20.19–26.54)	**24.14** *1.77* (20.13–27.56)	**22.81** *2.25* *(17.78–29.35)*	**23.50** *1.63* (19.13–27.03)	0.000**	0.002**	0.034*
	R	**25.25** *3.71* (18.40–33.88)	**24.72** *2.08* (19.72–30.31)	**23.45** *1.84* (19.58–27.19)	**24.70** *1.69* (21.18–28.75)		0.028*	0.020*

*Note*: Bold: mean, Italics: standard deviation, Brackets indicates the minimum and maximum values. Statistically significant *p ≤ 0.05, **p ≤ 0.005.

**TABLE 6:** Variation in ocular position and protrusion between sexes within populations and between populations within sexes.

Measurement	Side	Sexual dimorphism within populations		Population variation within sex groups	
		Black south Africans *n* = 94	White south Africans *n* = 103	SA females *n* = 100	SA males *n* = 97
SOM‐os	L	0.007*	0.560	0.000**	0.021
	R	0.000**	0.868	0.000**	0.006
IOM‐oi	L	0.999	0.137	0.001**	0.426
	R	0.878	0.188	0.001**	0.980
MOM‐om	L	0.549	0.000**	0.842	0.000**
	R	0.000**	0.001**	0.044*	0.247
LOM (ek)–ol	L	0.016*	0.000**	0.000**	0.000**
	R	0.009*	0.000**	0.921	0.000**
LOM (dLOM)–ol	L	0.943	0.033*	0.019*	0.987
	R	0.665	0.000**	0.001**	0.293
Protrusion: (dLOM—oa)	L	0.457	0.000**	0.667	0.470
	R	0.986	0.000**	0.006*	0.813
SOM‐oa	L	0.725	0.999	0.001**	0.039*
	R	0.999	0.999	0.013*	0.011*
IOM‐oa	L	0.127	0.985	0.759	0.605
	R	0.332	0.982	0.879	0.162
MOM‐oa	L	0.721	0.000**	0.527	0.011*
	R	0.078	0.010*	0.004**	0.455
LOM (ek)‐oa	L	0.788	0.237	0.044*	0.312
	R	0.856	0.003**	0.005**	0.999

*Note*: Statistically significant *p ≤ 0.05, **p ≤ 0.005.

The eyeballs of Black South Africans protruded more from the orbital margins, with significant variation observed at the superior and lateral (ectoconchion) orbital margins, which appear to be sex‐specific (Table 6). Although Black South African females presented with significantly smaller ocular and orbital dimensions than White South African females, protrusion values were greater in this group, specifically with regard to the superior, medial and lateral orbital margins. Less variation was observed in the male sample. No sexual dimorphism was noted in the protrusion values of the Black South African sample, while the eyeballs of White males protruded significantly further from the deepest point of the lateral orbital wall compared to White females.

## 4 DISCUSSION

This study assessed the ocular and orbital dimensions in order to establish the size and position of the eye in the bony orbit in a South African sample as it is integral in facial approximations [2, 9, 10]. Side, sex, and population affinity had an influence on the ocular and orbital dimensions as well as the eyeball position. Subtle variations in orbital dimensions and eyeball positioning could cumulatively influence the accuracy of facial recognition based on the generated forensic approximation [5, 21].

Rigid reproducibility testing was performed using two statistical tests, a mean dispersion analysis and an intraclass correlation coefficient test, to ensure reliable results. Hard‐tissue landmarks could be placed with greater accuracy, reflected in the mathematically calculated dimensions. Poorer repeatability was noted for the soft tissue landmarks placed on the equator of the eyeball for eyeball size determination. This was not unexpected as Casselman and colleagues (2013) [59] as well as Dorfling and co‐workers (2018) [22] highlight the difficulty in visualizing soft tissue structures on CBCT scans, which hampers accurate landmark placement. As the scans were from patients with open eyes, it was easier to locate the oculus anterius which was placed with great accuracy, as depicted by the relatively low dispersion analysis results (intra‐observer: 0.66 mm; interobserver: 0.77 mm). This resulted in higher ICC values for the distances representing the protrusion of the eyeball (Table 2).

The effect of sexual dimorphism and population affinity on the dimensions and shape of the cranium, including the orbital region, have been described in the literature [60, 61, 62, 63, 64, 65, 66, 67, 68, 69, 70, 71, 72]. In this South African sample, Black females presented with the smallest orbital dimensions when compared to the other South African groups, while the orbital index of White South African females was the greatest, which indicates a squarer orbit compared to a rectangular orbit (in the horizontal plane) noted in the other South African groups. White males displayed the largest orbital dimensions, which correspond with the literature describing greater cranial dimensions in this group when compared to other South African sex‐population groups [73, 74, 75]. A Bayes Factor calculation facilitated a direct statistical comparison of our results to previously published literature. In contrast with the rectangular orbits in the horizontal plane, noted in this South African sample, statistically significant differences were noted when compared to Egyptian [76] and Iranian [77] males and females (BF >2) who present with a rectangular orbit in the vertical plane.

The orbital height of White South African females corresponded to the orbital height reported in a Turkish (BF: −0.572) [40] and Korean [42] (BF: −0.313) sample, while the orbital dimensions of White South African females were significantly greater than Chinese [36] (BF: 12.213); White American [37] (BF: 8.399), French [39] (BF: 4.720) and Italian [78] (BF: 4.178) females. Orbital dimensions, and more specifically orbital height, in Black South African females, were more comparable to other female groups of French [39] (BF: −0.652), Korean [79] (BF: −0.612), Egyptian [76] (BF: −0.647) and Iranian [77] (BF: −0.233) decent. The orbital dimensions of South African males resembled Turkish [40] (BF: −0.738), Korean [42] (BF: −0.569), and Iranian [77] (BF: −0.727) males, although significantly greater dimensions were noted when compared to Chinese [36] (BF: 13.440), White American [37] (BF: 10.305), French [39] (BF: 4.735), Italian [78] (BF: 5.529) and Japanese [80] (BF: 2.269) males. Sexual dimorphism in the orbital dimensions, with larger dimensions noted in males, was not only observed in the current study but has been reported previously [36, 39, 40, 42, 80].

In contrast to the variations noted in the orbital dimensions of South Africans, no sexual dimorphism was observed in the ocular dimensions of White South Africans, which translates to relatively larger eyeballs in White females, located in generally smaller orbits. The cause of this sex‐related difference in White South Africans remains unclear, but may merely serve a functional purpose, such as maintaining optimal optical refraction. However, this trend was not observed in Black females, as all ocular measurements were significantly smaller compared to the other South African groups. In general, the ocular dimensions of South Africans were smaller in all planes when compared to the literature [22, 79, 81] (BF >2.000 in all groups), although the ocular axial length of South African males and White South African females was similar to Turkish [40] (male BF: −0.744; female BF: −0.680) and European [7] (male BF: −0.448; female BF: −0.476) samples. These variations emphasize the necessity for the use of sex‐ and population‐specific eyeballs during the facial approximation process, rather than the standard‐sized eyeballs with a 25 mm diameter currently used [12, 13, 14].

Interpopulation variation in normative orbital and ocular dimensions within this South African sample influenced the position of the eyeball within the orbit. The eyeball was often located deeper within the orbit in White South Africans compared to Black South Africans. More protruding eyeballs in the vertical and horizontal planes were noted in Black South African females when compared to White South African females, regardless of the significantly smaller orbital and ocular dimensions noted in Black South African females. These findings support the shallower position of the eyeball of Black South African females in relation to the superior orbital margin. Less variation was noted in protrusion values between South African males, although the eye protruded more from the superior orbital margin in Black South African males. These variations should be considered during facial approximations of South African faces.

Stephan and colleagues, 2009 [21] concluded that the eye was located closer to the superior and lateral orbital walls, with only 2/13 cadavers showing central positioning. The mean distances from the lateral orbital margin (dLOM) to the oculus laterale (ol) also indicated that South Africans' eyes are closer to the lateral orbital margin than the medial orbital margin. The distance from the superior orbital margin (sk) to the oculus superius (os) was however greater compared to the distance between the inferior orbital margin (or) and oculus inferius (oi) in this South African sample. The increased distance could be due to the more projecting superior orbital margin in relation to the inferior orbital margin [37], leading to an increased calculated distance, which cannot be compared to the studies performed by Stephan et al. (2009) [21] or Dorfling et al. (2018) [22] who calculated the position of the eye to the closest orbital wall, rather than the orbital margin.

The eyeball of South Africans protruded more from the deepest point of the lateral orbital margin compared to studies conducted on Australian [21], French [39], Black, and White Americans [44] as well as Japanese adults [80] (BF: >2.000). However, protrusion with regard to the dacryon was only 2–3 mm greater in South Africans. The significant variations noted between these studies could be ascribed to population variation, but gravitational effects on the CT and cadaveric tissue, the small sample size [21], and the average values representing males and females should be noted. Another factor that should be investigated further is the effect of nutritional status and body mass index (BMI) on the position and protrusion of the eyeball from the orbital rim, as it was reported to lead to increased protrusion values in a Japanese sample [80, 82]. Sexual dimorphism is commonly observed in ocular protrusion regardless of the method or modality used. As in this study, males in general, present with larger protrusion values compared to their female counterparts [39, 44].

The effect of gravity on the soft tissue structure of the face has been investigated. Martin and colleagues (2015) noticed that gravity influences the soft tissue structure of the face and indicated a maximum extension strain of up to 15% in the infraorbital region between the upright and supine position [83]. In a more recent study by Munn and Stephan (2018) based on high‐resolution dimensional imaging stereo‐photographs, the inferolateral soft tissue covering the orbit retracts laterally in the supine position [25]. However, the effect of gravity on the position of the eyeball has not been quantified.

## 5 CONCLUSION

This study emphasizes that variations in the average dimensions of the eye and orbit exist between sex and population groups which directly affect the eye's position in the orbit. These findings highlight the need for the creation and use of sex‐ and population‐specific guidelines to produce accurate facial approximations of South Africans. For Black South African females, given their significantly smaller eyeballs compared to other South African groups, an eyeball size of 21.98 × 22.44 × 23.77 mm is recommended, while the normative ocular size of White South African females is 23.50 × 23.23 × 23.88 mm. Due to the similarity in the ocular dimensions of South African males, a mean eyeball size of 23.21 × 23.45 × 24.92 mm should be considered. The use of CBCT scans to establish ocular position and protrusion is valuable, as the effect of gravity can be negated while more precise measurements can be obtained from superior image resolution compared to CT scans [22, 59, 84].

## CONFLICT OF INTEREST STATEMENT

The authors declare that they have no competing interests to disclose.

## Supporting information


Table S1.
