# Supplementary material for: Dimensions and position of the eye for facial approximations in a South African cone beam computed tomography sample
Source: J Forensic Sci. 2024 Dec 23;70(2):446–59. doi: 10.1111/1556-4029.15693 (PMC11874133; doi:10.1111/1556-4029.15693)
Supplement: Supplementary file 1 — Table S1. [file JFO-70-446-s001.docx]

| TABLE S1 Results of Bayes factor for statistical comparison of data to existing literature. | | | | | | | | |
| --- | --- | --- | --- | --- | --- | --- | --- | --- |
| **Linear distance / dimension** | **This study** | **Mean ± SD** | **Author** | **Sex (sample size)** | **Population** | **Modality** | **Mean ± SD** | **Bayes Factor** |
|  | **Sex (sample size)** |  |  |  |  |  |  | **Log 10 value*** |
| Left Orbital height | Black South African Female  (n = 45) | 35.73 ± 2.98 | Current study, 2024 | F (n = 55) | White South African | CBCT | 37.32 ± 2.28 | 0.955 |
|  |  |  | Özer et al., 2016 | F (n = 83) | Turkish | CT | 36.97 ± 2.19 | 0.458 |
|  |  |  | Attia et al., 2018 | F (n = 48) | Egyptian | CT | 35.90 ± 2.80 | -0.647 |
|  |  |  | Kim et al., 2016 | F (n = 50) | Korean | CBCT | 37.90 ± 2.00 | 2.421 |
|  |  |  | Weaver et al., 2010 | F (n = 23) | White American | CT | 31.75 ± 2.51 | 3.687 |
|  |  |  | Ji et al., 2010 | F (n = 34) | Chinese | CT | 33.22 ± 1.73 | 3.188 |
|  |  |  | Guyomarch et al., 2012 | F (n = 171) | French | CT | 35.40 ± 2.45 | -0.652 |
|  |  |  | Khani et al., 2023 | F (n = 51) | Iranian | CT | 36.70 ± 1.80 | -0.233 |
|  |  |  | Shin et al., 2016 | F (n = 72) | Korean | CT | 35.40 ± 2.00 | -0.612 |
|  |  |  | Cappella et al., 2020 | F (n = 40) | Italian | Skulls | 35.00 ± 2.00 | -0.306 |
|  |  |  | Kazuta et al., 2022 | F (n = 61) | Japanese | CT (cadaver) | 38.38 ± 2.60 | 3.441 |
|  | White South African Female  (n = 55) | 37.32 ± 2.28 | Current study, 2024 | F (n = 45) | Black South African | CBCT | 35.73 ± 2.98 | 0.955 |
|  |  |  | Özer et al., 2016 | F (n = 83) | Turkish | CT | 36.97 ± 2.19 | -0.572 |
|  |  |  | Attia et al., 2018 | F (n = 48) | Egyptian | CT | 35.90 ± 2.80 | 0.800 |
|  |  |  | Kim et al., 2016 | F (n = 50) | Korean | CBCT | 37.90 ± 2.00 | -0.313 |
|  |  |  | Weaver et al., 2010 | F (n = 23) | White American | CT | 31.75 ± 2.51 | 8.399 |
|  |  |  | Ji et al., 2010 | F (n = 34) | Chinese | CT | 33.22 ± 1.73 | 12.213 |
|  |  |  | Guyomarch et al., 2012 | F (n = 171) | French | CT | 35.40 ± 2.45 | 4.720 |
|  |  |  | Khani et al., 2023 | F (n = 51) | Iranian | CT | 36.70 ± 1.80 | 0.130 |
|  |  |  | Shin et al., 2016 | F (n = 72) | Korean | CT | 35.40 ± 2.00 | 3.819 |
|  |  |  | Cappella et al., 2020 | F (n = 40) | Italian | Skulls | 35.00 ± 2.00 | 4.178 |
|  |  |  | Kazuta et al., 2022 | F (n = 61) | Japanese | CT (cadaver) | 38.38 ± 2.60 | 0.352 |

| Left Orbital height (cont) | Black South African Males  (n = 49) | 37.79 ± 2.28 | Current study, 2024 | M (n = 48) | White South African | CBCT | 38.05 ± 2.15 | -0.590 |
| --- | --- | --- | --- | --- | --- | --- | --- | --- |
|  |  |  | Özer et al., 2016 | M (n = 115) | Turkish | CT | 37.77 ± 2.48 | -0.738 |
|  |  |  | Attia et al., 2018 | M (n = 44) | Egyptian | CT | 36.90 ± 2.90 | -0.188 |
|  |  |  | Kim et al., 2016 | M (n = 50) | Korean | CBCT | 38.10 ± 2.30 | -0.569 |
|  |  |  | Weaver et al., 2010 | M (n = 16) | White American | CT | 32.44 ± 1.89 | 10.305 |
|  |  |  | Ji et al., 2010 | M (n = 30) | Chinese | CT | 33.35 ± 1.44 | 13.440 |
|  |  |  | Guyomarch et al., 2012 | M (n = 204) | French | CT | 35.80 ± 2.45 | 4.735 |
|  |  |  | Khani et al., 2023 | M (n = 100) | Iranian | CT | 37.80 ± 2.00 | -0.727 |
|  |  |  | Shin et al., 2016 | M (n = 102) | Korean | CT | 35.60 ± 1.90 | 5.344 |
|  |  |  | Cappella et al., 2020 | M (n = 40) | Italian | Skulls | 33.63 ± 2.00 | 5.529 |
|  |  |  | Kazuta et al., 2022 | M (n = 85) | Japanese | CT (cadaver) | 39.42 ± 2.42 | 2.269 |
|  | White South African Males  (n = 48) | 38.05 ± 2.15 | Current study, 2024 | M (n = 52) | Black South African | CBCT | 37.79 ± 2.28 | -0.590 |
|  |  |  | Özer et al., 2016 | M (n = 115) | Turkish | CT | 37.77 ± 2.48 | -0.631 |
|  |  |  | Attia et al., 2018 | M (n = 44) | Egyptian | CT | 36.90 ± 2.90 | 0.210 |
|  |  |  | Kim et al., 2016 | M (n = 50) | Korean | CBCT | 38.10 ± 2.30 | -0.670 |
|  |  |  | Weaver et al., 2010 | M (n = 16) | White American | CT | 32.44 ± 1.89 | 11.301 |
|  |  |  | Ji et al., 2010 | M (n = 30) | Chinese | CT | 33.35 ± 1.44 | 15.201 |
|  |  |  | Guyomarch et al., 2012 | M (n = 204) | French | CT | 35.80 ± 2.45 | 6.922 |
|  |  |  | Khani et al., 2023 | M (n = 100) | Iranian | CT | 37.80 ± 2.00 | -0.636 |
|  |  |  | Shin et al., 2016 | M (n = 102) | Korean | CT | 35.60 ± 1.90 | 7.418 |
|  |  |  | Cappella et al., 2020 | M (n = 40) | Italian | Skulls | 33.63 ± 2.00 | 6.967 |
|  |  |  | Kazuta et al., 2022 | M (n = 85) | Japanese | CT (cadaver) | 39.42 ± 2.42 | 1.461 |
| Left Orbital breadth | Black South African Female  (n = 45) | 40.10 ± 2.79 | Current study, 2024 | F (n = 57) | White South African | CBCT | 40.52 ± 1.45 | -0.513 |
|  |  |  | Özer et al., 2016 | F (n = 83) | Turkish | CT | 33.27 ± 1.77 | 26.086 |
|  |  |  | Attia et al., 2018 | F (n = 48) | Egyptian | CT | 37.00 ± 3.60 | 3.204 |
|  |  |  | Kim et al., 2016 | F (n = 50) | Korean | CBCT | 35.90 ± 2.80 | 7.916 |
|  |  |  | Weaver et al., 2010 | F (n = 23) | White American | CT | 36.60 ± 1.71 | 4.704 |
|  |  |  | Ji et al., 2010 | F (n = 34) | Chinese | CT | 38.00 ± 2.25 | 1.822 |
|  |  |  | Guyomarch et al., 2012 | F (n = 171) | French | CT | 37.80 ± 1.98 | 4.437 |
|  |  |  | Khani et al., 2023 | F (n = 51) | Iranian | CT | 34.60 ± 1.60 | 16.701 |
|  |  |  | Shin et al., 2016 | F (n = 72) | Korean | CT | 44.50 ± 1.80 | 1.476 |
|  |  |  | Cappella et al., 2020 | F (n = 40) | Italian | Skulls | 39.00 ± 2.00 | 2.253 |
|  |  |  | Kazuta et al., 2022 | F (n = 61) | Japanese | CT (cadaver) | 40.78 ± 2.73 | -0.380 |
| Left orbital breadth (cont) | White South African Female  (n = 55) | 40.52 ± 1.45 | Current study, 2024 | F (n = 57) | Black South African | CBCT | 40.10 ± 2.79 | -0.513 |
|  |  |  | Özer et al., 2016 | F (n = 83) | Turkish | CT | 33.27 ± 1.77 | 51.398 |
|  |  |  | Attia et al., 2018 | F (n = 48) | Egyptian | CT | 37.00 ± 3.60 | 6.171 |
|  |  |  | Kim et al., 2016 | F (n = 50) | Korean | CBCT | 35.90 ± 2.80 | 14.734 |
|  |  |  | Weaver et al., 2010 | F (n = 23) | White American | CT | 36.60 ± 1.71 | 9.035 |
|  |  |  | Ji et al., 2010 | F (n = 34) | Chinese | CT | 38.00 ± 2.25 | 5.081 |
|  |  |  | Guyomarch et al., 2012 | F (n = 171) | French | CT | 37.80 ± 1.98 | 19.517 |
|  |  |  | Khani et al., 2023 | F (n = 51) | Iranian | CT | 34.60 ± 1.60 | 33.675 |
|  |  |  | Shin et al., 2016 | F (n = 72) | Korean | CT | 44.50 ± 1.80 | 2.263 |
|  |  |  | Cappella et al., 2020 | F (n = 40) | Italian | Skulls | 39.00 ± 2.00 | 6.888 |
|  |  |  | Kazuta et al., 2022 | F (n = 61) | Japanese | CT (cadaver) | 40.78 ± 2.73 | -0.622 |
|  | Black South African Males  (n = 49) | 42.27 ± 1.62 | Current study, 2024 | M (n = 52) | White South African | CBCT | 42.76 ± 1.50 | -0.213 |
|  |  |  | Özer et al., 2016 | M (n = 115) | Turkish | CT | 34.17 ± 2.10 | 57.393 |
|  |  |  | Attia et al., 2018 | M (n = 44) | Egyptian | CT | 36.70 ± 4.10 | 10.081 |
|  |  |  | Kim et al., 2016 | M (n = 50) | Korean | CBCT | 42.10 ± 1.80 | -0.627 |
|  |  |  | Weaver et al., 2010 | M (n = 16) | White American | CT | 37.42 ± 2.44 | 7.282 |
|  |  |  | Ji et al., 2010 | M (n = 30) | Chinese | CT | 40.02 ± 1.63 | 5.198 |
|  |  |  | Guyomarch et al., 2012 | M (n = 204) | French | CT | 39.50 ± 1.98 | 17.652 |
|  |  |  | Khani et al., 2023 | M (n = 100) | Iranian | CT | 35.00 ± 1.60 | 52.711 |
|  |  |  | Shin et al., 2016 | M (n = 102) | Korean | CT | 41.70 ± 1.90 | 9.404 |
|  |  |  | Cappella et al., 2020 | M (n = 40) | Italian | Skulls | 38.00 ± 2.00 | 9.769 |
|  |  |  | Kazuta et al., 2022 | M (n = 85) | Japanese | CT (cadaver) | 42.94 ± 2.57 | -0.050 |
|  | White South African Males  (n = 48) | 42.76 ± 1.50 | Current study, 2024 | M (n = 52) | Black South African | CBCT | 42.27 ± 1.62 | -0.213 |
|  |  |  | Özer et al., 2016 | M (n = 115) | Turkish | CT | 34.17 ± 2.10 | 64.753 |
|  |  |  | Attia et al., 2018 | M (n = 44) | Egyptian | CT | 36.70 ± 4.10 | 11.670 |
|  |  |  | Kim et al., 2016 | M (n = 50) | Korean | CBCT | 42.10 ± 1.80 | 0.072 |
|  |  |  | Weaver et al., 2010 | M (n = 16) | White American | CT | 37.42 ± 2.44 | 8.579 |
|  |  |  | Ji et al., 2010 | M (n = 30) | Chinese | CT | 40.02 ± 1.63 | 7.753 |
|  |  |  | Guyomarch et al., 2012 | M (n = 204) | French | CT | 39.50 ± 1.98 | 25.367 |
|  |  |  | Khani et al., 2023 | M (n = 100) | Iranian | CT | 35.00 ± 1.60 | 58.257 |
|  |  |  | Shin et al., 2016 | M (n = 102) | Korean | CT | 41.70 ± 1.90 | 6.265 |
|  |  |  | Cappella et al., 2020 | M (n = 40) | Italian | Skulls | 38.00 ± 2.00 | 12.594 |
|  |  |  | Kazuta et al., 2022 | M (n = 85) | Japanese | CT (cadaver) | 42.94 ± 2.57 | -0.665 |
| Left Orbital Index | Black South African Females  (n = 45) | 89.17 ± 5.09 | Current study, 2024 | F (n = 57) | White South African | CBCT | 92.19 ± 6.20 | 0.705 |
|  |  |  | Attia et al., 2018 | F (n = 48) | Egyptian | CT | 97.90 ± 10.60 | 3.950 |
|  |  |  | Khani et al., 2023 | F (n = 51) | Iranian | CT | 108.59 ± 6.03 | 27.186 |
|  | White South African Females (n = 55) | 92.19 ± 6.20 | Current study, 2024 | F (n = 45) | Black South African | CBCT | 89.19 ± 5.09 | 0.705 |
|  |  |  | Attia et al., 2018 | F (n = 48) | Egyptian | CT | 97.90 ± 10.60 | 1.331 |
|  |  |  | Khani et al., 2023 | F (n = 51) | Iranian | CT | 108.59 ± 6.03 | 21.896 |
|  | Black South African Males  (n = 49) | 89.36 ± 5.08 | Current study, 2024 | M (n = 52) | White South African | CBCT | 89.03 ± 4.66 | -0.649 |
|  |  |  | Attia et al., 2018 | M (n = 44) | Egyptian | CT | 101.40 ± 11.40 | 6.658 |
|  |  |  | Khani et al., 2023 | M (n = 100) | Iranian | CT | 108.25 ±7.38 | 37.557 |
|  | White South African Males  (n = 48) | 89.03 ± 4.66 | Current study, 2024 | M (n = 52) | Black South African | CBCT | 89.36 ± 5.08 | -0.649 |
|  |  |  | Attia et al., 2018 | M (n = 44) | Egyptian | CT | 101.40 ± 11.40 | 6.053 |
|  |  |  | Khani et al., 2023 | M (n = 100) | Iranian | CT | 108.25 ±7.38 | 36.906 |
| Left ocular height | Black South African Females  (n = 45) | 23.77 ± 1.44 | Current study, 2024 | F (n = 57) | White South African | CBCT | 23.88 ± 1.43 | -0.646 |
|  |  |  | Shin et al., 2016 | F (n = 72) | Korean | CT | 26.60 ± 1.80 | 12.784 |
|  | White South African Females (n = 55) | 23.88 ± 1.43 | Current study, 2024 | F (n = 45) | Black South African | CBCT | 23.77 ± 1.44 | -0.646 |
|  |  |  | Shin et al., 2016 | F (n = 72) | Korean | CT | 26.60 ± 1.80 | 6.037 |
|  | Black South African Males (n = 49) | 24.60 ± 1.60 | Current study, 2024 | M (n = 52) | White South African | CBCT | 25.23 ± 1.55 | -0.042 |
|  |  |  | Shin et al., 2016 | M (n = 102) | Korean | CT | 26.50 ± 1.80 | 14.656 |
|  | White South African Males (n = 48) | 25.23 ± 1.55 | Current study, 2024 | M (n = 52) | Black South African | CBCT | 24.60 ± 1.60 | -0.042 |
|  |  |  | Shin et al., 2016 | M (n = 102) | Korean | CT | 26.50 ± 1.80 | 3.193 |
| Left Ocular breadth | Black South African Females  (n = 45) | 22.44 ± 1.60 | Current study, 2024 | F (n = 57) | White South African | CBCT | 23.23 ± 1.20 | 0.744 |
|  |  |  | Shin et al., 2016 | F (n = 72) | Korean | CT | 25.70 ± 1.30 | 15.039 |
|  |  |  | Rana et al., 2022 | F (n = 111) | Australian | MRI | 25.20 ± 1.00 | 20.898 |
|  | White South African Females  (n = 55) | 23.23 ± 1.20 | Current study, 2024 | F (n = 45) | Black South African | CBCT | 22.44 ± 1.60 | 0.744 |
|  |  |  | Shin et al., 2016 | F (n = 72) | Korean | CT | 25.70 ± 1.30 | 14.156 |
|  |  |  | Rana et al., 2022 | F (n = 111) | Australian | MRI | 25.20 ± 1.00 | 21.291 |

| Left Ocular breadth (cont) | Black South African Males (n = 49) | 23.34 ± 1.47 | Current study, 2024 | M (n = 52) | White South African | CBCT | 23.55 ± 1.39 | -0.570 |
| --- | --- | --- | --- | --- | --- | --- | --- | --- |
|  |  |  | Shin et al., 2016 | M (n = 102) | Korean | CT | 26.20 ± 1.30 | 16.704 |
|  |  |  | Rana et al., 2022 | M (n = 90) | Australian | MRI | 25.80 ± 1.00 | 18.353 |
|  | White South African Males (n = 48) | 23.55 ± 1.39 | Current study, 2024 | M (n = 52) | Black South African | CBCT | 23.34 ± 1.47 | -0.570 |
|  |  |  | Shin et al., 2016 | M (n = 102) | Korean | CT | 26.20 ± 1.30 | 15.300 |
|  |  |  | Rana et al., 2022 | M (n = 90) | Australian | MRI | 25.80 ± 1.00 | 17.077 |
| Left ocular / axial length | Black South African Females  (n = 45) | 21.98 ± 1.23 | Current study, 2024 | F (n = 57) | White South African | CBCT | 23.50 ± 1.22 | 3.170 |
|  |  |  | Özer et al., 2016 | F (n = 83) | Turkish | CT | 22.76 ± 6.38 | -0.479 |
|  |  |  | Wilkinson and Mautner, 2003 | F (n = 28) | UK | MRI | 23.42 ± 1.41 | 2.793 |
|  |  |  | Rana et al., 2022 | F (n = 111) | Australian | MRI | 24.70 ± 0.95 | 24.001 |
|  |  |  | Shin et al., 2016 | F (n = 72) | Korean | CT | 26.10 ± 1.10 | 32.391 |
|  | White South African Females  (n = 55) | 23.50 ± 1.22 | Current study, 2024 | F (n = 45) | Black South African | CBCT | 21.98 ± 1.23 | 3.170 |
|  |  |  | Özer et al., 2016 | F (n = 83) | Turkish | CT | 22.76 ± 6.38 | -0.680 |
|  |  |  | Wilkinson and Mautner, 2003 | F (n = 28) | UK | MRI | 23.42 ± 1.41 | -0.476 |
|  |  |  | Rana et al., 2022 | F (n = 111) | Australian | MRI | 24.70 ± 0.95 | 10.522 |
|  |  |  | Shin et al., 2016 | F (n = 72) | Korean | CT | 26.10 ± 1.10 | 23.502 |
|  | Black South African Males  (n = 49) | 23.14 ± 1.30 | Current study, 2024 | M (n = 52) | White South African | CBCT | 23.27 ± 1.17 | -0.497 |
|  |  |  | Özer et al., 2016 | M (n = 115) | Turkish | CT | 23.25 ± 0.88 | -0.399 |
|  |  |  | Wilkinson and Mautner, 2003 | M (n = 11) | UK | MRI | 23.21 ± 1.76 | -0.448 |
|  |  |  | Rana et al., 2022 | M (n = 90) | Australian | MRI | 25.20 ± 1.10 | 10.673 |
|  |  |  | Shin et al., 2016 | M (n = 102) | Korean | CT | 26.60 ± 1.10 | 29.028 |
|  | White South African Males  (n = 48) | 23.27 ± 1.17 | Current study, 2024 | M (n = 52) | Black South African | CBCT | 23.14 ± 1.30 | -0.497 |
|  |  |  | Özer et al., 2016 | M (n = 115) | Turkish | CT | 23.25 ± 0.88 | -0.744 |
|  |  |  | Wilkinson and Mautner, 2003 | M (n = 11) | UK | MRI | 23.21 ± 1.76 | -0.492 |
|  |  |  | Rana et al., 2022 | M (n = 90) | Australian | MRI | 25.20 ± 1.10 | 14.163 |
|  |  |  | Shin et al., 2016 | M (n = 102) | Korean | CT | 26.60 ± 1.10 | 33.531 |

| Left eye protrusion from LOM (dLOM –oa) | Black South African Females  (n = 45) | 24.02 ± 2.26 | Current study, 2024 | F (n = 55) | White South African | CBCT | 23.59 ± 1.61 | -0.453 |
| --- | --- | --- | --- | --- | --- | --- | --- | --- |
|  |  |  | Guyomarch et al., 2012 | F (n = 171) | French | CT | 17.60 ± 2.32 | 37.513 |
|  |  |  | Barretto and Mathog, 1999 | F (n = 28) | Black American | Patients | 17.27 ± 1.41 | 21.412 |
|  |  |  | Barretto and Mathog, 1999 | F (n = 31) | White American | Patients | 16.02 ± 2.23 | 21.214 |
|  |  |  | Dunsky, 1992 | F (n = 170) | Black American | Patients | 17.46 ± 2.64 | 36.921 |
|  |  |  | Kim et al., 2016 | F (n = 50) | Korean | CBCT | 16.40 ± 1.30 | 31.641 |
|  |  |  | Kazuta et al., 2022 | F (n = 61) | Japanese | CT (cadaver) | 14.17 ± 3.52 | 29.262 |
|  | White South African Females  (n = 55) | 23.59 ± 1.61 | Current study, 2024 | F (n = 45) | Black South African | CBCT | 24.02 ± 2.26 | -0.453 |
|  |  |  | Guyomarch et al., 2012 | F (n = 171) | French | CT | 17.60 ± 2.32 | 52.174 |
|  |  |  | Barretto and Mathog, 1999 | F (n = 28) | Black American | Patients | 17.27 ± 1.41 | 27.121 |
|  |  |  | Barretto and Mathog, 1999 | F (n = 31) | White American | Patients | 16.02 ± 2.23 | 24.819 |
|  |  |  | Dunsky, 1992 | F (n = 170) | Black American | Patients | 17.46 ± 2.64 | 49.906 |
|  |  |  | Kim et al., 2016 | F (n = 50) | Korean | CBCT | 16.40 ± 1.30 | 42.239 |
|  |  |  | Kazuta et al., 2022 | F (n = 61) | Japanese | CT (cadaver) | 14.17 ± 3.52 | 33.220 |
|  | Black South African Males  (n = 49) | 24.60 ± 1.91 | Current study, 2024 | M (n = 48) | White South African | CBCT | 25.16 ± 1.81 | -0.249 |
|  |  |  | Guyomarch et al., 2012 | M (n = 204) | French | CT | 18.70 ± 2.32 | 45.334 |
|  |  |  | Barretto and Mathog, 1999 | M (n = 33) | Black American | Patients | 18.23 ±2.19 | 19.162 |
|  |  |  | Barretto and Mathog, 1999 | M (n = 34) | White American | Patients | 17.01 ± 2.70 | 20.205 |
|  |  |  | Dunsky, 1992 | M (n = 139) | Black American | Patients | 18.20 ± 2.97 | 36.788 |
|  |  |  | Kim et al., 2016 | M (n = 50) | Korean | CBCT | 17.10 ± 1.30 | 37.088 |
|  |  |  | Kazuta et al., 2022 | M (n = 85) | Japanese | CT (cadaver) | 15.40 ± 3.62 | 36.501 |
|  | White South African Males  (n = 48) | 25.16 ± 1.81 | Current study, 2024 | M (n = 49) | Black South African | CBCT | 24.60 ± 1.91 | -0.249 |
|  |  |  | Guyomarch et al., 2012 | M (n = 204) | French | CT | 18.70 ± 2.32 | 53.298 |
|  |  |  | Barretto and Mathog, 1999 | M (n = 33) | Black American | Patients | 18.23 ±2.19 | 21.438 |
|  |  |  | Barretto and Mathog, 1999 | M (n = 34) | White American | Patients | 17.01 ± 2.70 | 22.128 |
|  |  |  | Dunsky, 1992 | M (n = 139) | Black American | Patients | 18.20 ± 2.97 | 42.157 |
|  |  |  | Kim et al., 2016 | M (n = 50) | Korean | CBCT | 17.10 ± 1.30 | 40.445 |
|  |  |  | Kazuta et al., 2022 | M (n = 85) | Japanese | CT (cadaver) | 15.40 ± 3.62 | 39.457 |

| Left eye protrusion from IOM | Black South African Females  (n = 45) | 20.51 ± 2.90 | Current study, 2024 | F (n = 55) | White South African | CBCT | 20.13 ± 1.80 | -0.561 |
| --- | --- | --- | --- | --- | --- | --- | --- | --- |
|  |  |  | Guyomarch et al., 2012 | F (n = 171) | French | CT | 19.70 ± 1.72 | -0.109 |
|  |  |  | Kim et al., 2016 | F (n = 50) | Korean | CBCT | 16.70 ± 1.70 | 8.675 |
|  | White South African Females  (n = 55) | 20.13 ± 1.80 | Current study, 2024 | F (n = 45) | Black South African | CBCT | 20.51 ± 2.90 | -0.561 |
|  |  |  | Guyomarch et al., 2012 | F (n = 171) | French | CT | 19.70 ± 1.72 | -0.290 |
|  |  |  | Kim et al., 2016 | F (n = 50) | Korean | CBCT | 16.70 ± 1.70 | 13.829 |
|  | Black South African Males  (n = 49) | 19.70 ± 1.99 | Current study, 2024 | M (n = 48) | White South African | CBCT | 20.42 ± 1.75 | 0.013 |
|  |  |  | Guyomarch et al., 2012 | M (n = 204) | French | CT | 19.90 ± 1.72 | -0.682 |
|  |  |  | Kim et al., 2016 | M (n = 50) | Korean | CBCT | 16.90 ± 1.40 | 9.531 |
|  | White South African Males  (n = 48) | 20.42 ± 1.75 | Current study, 2024 | M (n = 49) | Black South African | CBCT | 19.70 ± 1.99 | 0.013 |
|  |  |  | Guyomarch et al., 2012 | M (n = 204) | French | CT | 19.90 ± 1.72 | -0.074 |
|  |  |  | Kim et al., 2016 | M (n = 50) | Korean | CBCT | 16.90 ± 1.40 | 15.445 |
| Left eye protrusion from MOM | Black South African Females  (n = 45) | 23.35 ± 2.47 | Current study, 2024 | F (n = 55) | White South African | CBCT | 22.81 ± 1.67 | -0.373 |
|  |  |  | Guyomarch et al., 2012 | F (n = 171) | French | CT | 21.70 ± 1.81 | 2.678 |
|  |  |  | Kim et al., 2016 | F (n = 50) | Korean | CBCT | 22.30 ± 1.40 | 0.524 |
|  | White South African Females  (n = 55) | 22.81 ± 1.67 | Current study, 2024 | F (n = 45) | Black South African | CBCT | 23.35 ± 2.47 | -0.373 |
|  |  |  | Guyomarch et al., 2012 | F (n = 171) | French | CT | 21.70 ± 1.81 | 2.687 |
|  |  |  | Kim et al., 2016 | F (n = 50) | Korean | CBCT | 22.30 ± 1.40 | -0.128 |
|  | Black South African Males  (n = 49) | 23.20 ± 1.97 | Current study, 2024 | M (n = 48) | White South African | CBCT | 24.33 ± 1.71 | 1.037 |
|  |  |  | Guyomarch et al., 2012 | M (n = 204) | French | CT | 22.80 ± 1.81 | -0.430 |
|  |  |  | Kim et al., 2016 | M (n = 50) | Korean | CBCT | 23.50 ± 1.70 | -0.547 |
|  | White South African Males  (n = 48) | 24.33 ± 1.71 | Current study, 2024 | M (n = 49) | Black South African | CBCT | 23.20 ± 1.97 | 1.037 |
|  |  |  | Guyomarch et al., 2012 | M (n = 204) | French | CT | 22.80 ± 1.81 | 5.117 |
|  |  |  | Kim et al., 2016 | M (n = 50) | Korean | CBCT | 23.50 ± 1.70 | 0.428 |
| Left eye protrusion from SOM | Black South African Females  (n = 45) | 21.31 ± 2.74 | Current study, 2024 | F (n = 55) | White South African | CBCT | 19.59 ± 2.06 | 1.606 |
|  |  |  | Guyomarch et al., 2012 | F (n = 171) | French | CT | 15.70 ± 1.91 | 25.229 |
|  |  |  | Kim et al., 2016 | F (n = 50) | Korean | CBCT | 20.70 ± 1.80 | -0.358 |
|  |  |  | Kazuta et al., 2022 | F (n = 61) | Japanese | CT (cadaver) | 20.64 ± 2.27 | -0.338 |
|  | White South African Females  (n = 55) | 19.59 ± 2.06 | Current study, 2024 | F (n = 45) | Black South African | CBCT | 21.31 ± 2.74 | 1.606 |
|  |  |  | Guyomarch et al., 2012 | F (n = 171) | French | CT | 15.70 ± 1.91 | 23.745 |
|  |  |  | Kim et al., 2016 | F (n = 50) | Korean | CBCT | 20.70 ± 1.80 | 0.986 |
|  |  |  | Kazuta et al., 2022 | F (n = 61) | Japanese | CT (cadaver) | 21.31± 2.74 | 0.607 |

| Left eye protrusion from SOM (cont) | Black South African Males  (n = 49) | 20.84 ± 2.04 | Current study, 2024 | M (n = 48) | White South African | CBCT | 19.65 ± 1.85 | 1.028 |
| --- | --- | --- | --- | --- | --- | --- | --- | --- |
|  |  |  | Guyomarch et al., 2012 | M (n = 204) | French | CT | 16.00 ± 1.91 | 33.508 |
|  |  |  | Kim et al., 2016 | M (n = 50) | Korean | CBCT | 20.50 ± 1.50 | -0.503 |
|  |  |  | Kazuta et al., 2022 | M (n = 85) | Japanese | CT (cadaver) | 22.24 ± 3.01 | 1.250 |
|  | White South African Males  (n = 48) | 19.65 ± 1.85 | Current study, 2024 | M (n = 49) | Black South African | CBCT | 20.84 ± 2.04 | 1.028 |
|  |  |  | Guyomarch et al., 2012 | M (n = 204) | French | CT | 16.00 ± 1.91 | 23.859 |
|  |  |  | Kim et al., 2016 | M (n = 50) | Korean | CBCT | 20.50 ± 1.50 | 0.504 |
|  |  |  | Kazuta et al., 2022 | M (n = 85) | Japanese | CT (cadaver) | 22.24 ± 3.01 | 6.037 |
| * Note: The Bayes Factor was interpreted based on Jeffreys' (1961) scale of the base 10 logarithmic Bayes Factor (logBF). The logBF provides evidence for the alternative hypothesis which posits variation between normative population means. The colours of the LogBF values can be interpreted as: | | | | | | | | |
|  | < -2 | Decisive evidence that there is no difference between population means | | | | | | |
|  | -2 < BF <-1,5 | Very strong evidence that there is no difference between population means | | | | | | |
|  | -1,5 < BF < -1 | Strong evidence that there is no difference between population means | | | | | | |
|  | -1 < BF < -0,5 | Substantial evidence that there is no difference between population means | | | | | | |
|  | -0,5 < BF < 0 | Weak evidence that there is no difference between population means | | | | | | |
|  | 0 < BF < 0,5 | Weak evidence that there is a difference between population means | | | | | | |
|  | 0,5 < BF < 1 | Substantial evidence that there is a difference between population means | | | | | | |
|  | 1 < BF < 1,5 | Strong evidence that there is a difference between population means | | | | | | |
|  | 1,5 < BF <2 | Very strong evidence that there is a difference between population means | | | | | | |
